# 1-(4-Bromo­phen­yl)-2-(2-chloro­phen­oxy)ethanone

**DOI:** 10.1107/S160053681204785X

**Published:** 2012-11-30

**Authors:** Seema S. Shenvi, Arun M. Isloor, Thomas Gerber, Eric Hosten, Richard Betz

**Affiliations:** a National Institute of Technology-Karnataka, Department of Chemistry, Surathkal, Mangalore 575 025, India; b Nelson Mandela Metropolitan University, Summerstrand Campus, Department of Chemistry, University Way, Summerstrand, PO Box 77000, Port Elizabeth, 6031, South Africa

## Abstract

In the title compound, C_14_H_10_BrClO_2_, a twofold halogenated derivative of phenyl­ated phenyl­oxyethanone, the least-squares planes defined by the C atoms of the aromatic rings subtend an angle of 71.31 (17)°. In the crystal, C—H⋯O contacts connect the mol­ecules into chains along the *b-*axis direction.

## Related literature

For the biological properties of phen­oxy­acetic acid derivatives, see: Ali & Shaharyar (2007 ▶); Kunsch *et al.* (2005 ▶); Iqbal *et al.* (2007 ▶); Sato *et al.* (2002 ▶); Kitagawa *et al.* (1991 ▶); Bicking *et al.* (1976 ▶); Osborne *et al.* (1955 ▶). For graph-set analysis of hydrogen bonds, see: Etter *et al.* (1990 ▶); Bernstein *et al.* (1995 ▶). For a description of the Cambridge Structural Database, see: Allen (2002 ▶).
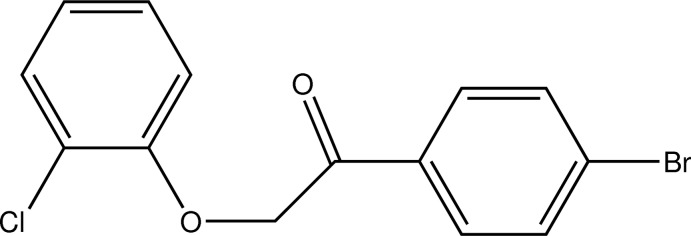


## Experimental

### 

#### Crystal data

C_14_H_10_BrClO_2_
*M* *_r_* = 325.58
Monoclinic, 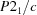

*a* = 15.2653 (8) Å
*b* = 4.5541 (2) Å
*c* = 23.7336 (9) Å
β = 129.135 (2)°
*V* = 1279.80 (10) Å^3^
*Z* = 4
Mo *K*α radiation
μ = 3.41 mm^−1^
*T* = 200 K
0.36 × 0.07 × 0.06 mm

#### Data collection

Bruker APEXII CCD diffractometer
Absorption correction: multi-scan (*SADABS*; Bruker, 2008 ▶) *T*_min_ = 0.377, *T*_max_ = 0.811
15447 measured reflections
3040 independent reflections
1953 reflections with *I* > 2σ(*I*)
*R*_int_ = 0.076

#### Refinement

*R*[*F* ^2^ > 2σ(*F* ^2^)] = 0.041
*wR*(*F* ^2^) = 0.125
*S* = 1.02
3040 reflections
163 parameters
H-atom parameters constrained
Δρ_max_ = 0.65 e Å^−3^
Δρ_min_ = −1.04 e Å^−3^

### 

Data collection: *APEX2* (Bruker, 2010 ▶); cell refinement: *SAINT* (Bruker, 2010 ▶); data reduction: *SAINT*; program(s) used to solve structure: *SHELXS97* (Sheldrick, 2008 ▶); program(s) used to refine structure: *SHELXL97* (Sheldrick, 2008 ▶); molecular graphics: *ORTEP-3* (Farrugia, 2012 ▶) and *Mercury* (Macrae *et al.*, 2008 ▶); software used to prepare material for publication: *SHELXL97* and *PLATON* (Spek, 2009 ▶).

## Supplementary Material

Crystal structure: contains datablock(s) I, global. DOI: 10.1107/S160053681204785X/rz5023sup1.cif

Supplementary material file. DOI: 10.1107/S160053681204785X/rz5023Isup2.cdx

Structure factors: contains datablock(s) I. DOI: 10.1107/S160053681204785X/rz5023Isup3.hkl

Supplementary material file. DOI: 10.1107/S160053681204785X/rz5023Isup4.cml

Additional supplementary materials:  crystallographic information; 3D view; checkCIF report

## Figures and Tables

**Table 1:** Hydrogen-bond geometry (Å, °)

*D*—H⋯*A*	*D*—H	H⋯*A*	*D*⋯*A*	*D*—H⋯*A*
C1—H1*B*⋯O2^i^	0.99	2.57	3.457 (4)	149
C16—H16⋯O2^i^	0.95	2.57	3.425 (4)	150

Symmetry code: (i) 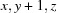.

**Fig. 1.**
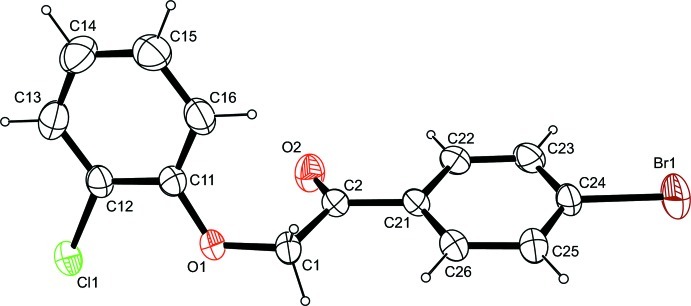
The molecular structure of the title compound, with atom labels and anisotropic displacement ellipsoids (drawn at 50% probability level).

**Fig. 2.**
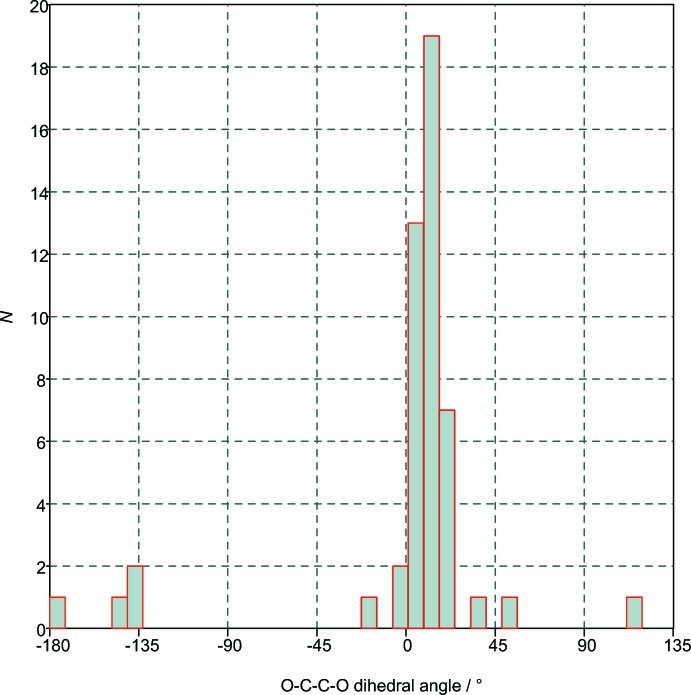
Observed distribution of O–C–C–O dihedral angles (data based on CSD search including all deposited crystal structures up to November 2011).

**Fig. 3.**
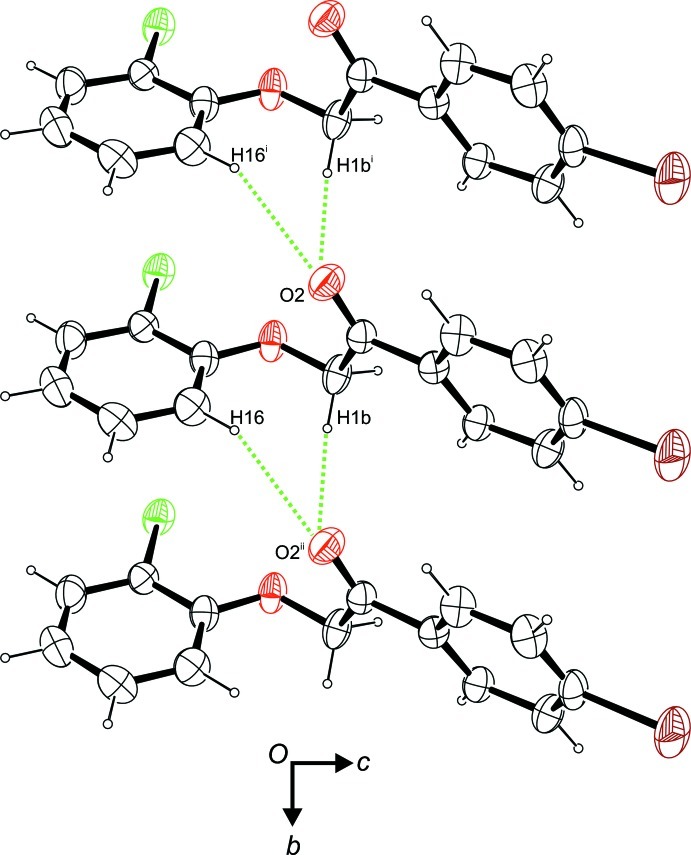
Intermolecular contacts, viewed along [-1 0 0]. Displacement ellipsoids are drawn at the 50% probability level. Symmetry operators: (i) x, y - 1, z; (ii) x, y + 1, z.

**Fig. 4.**
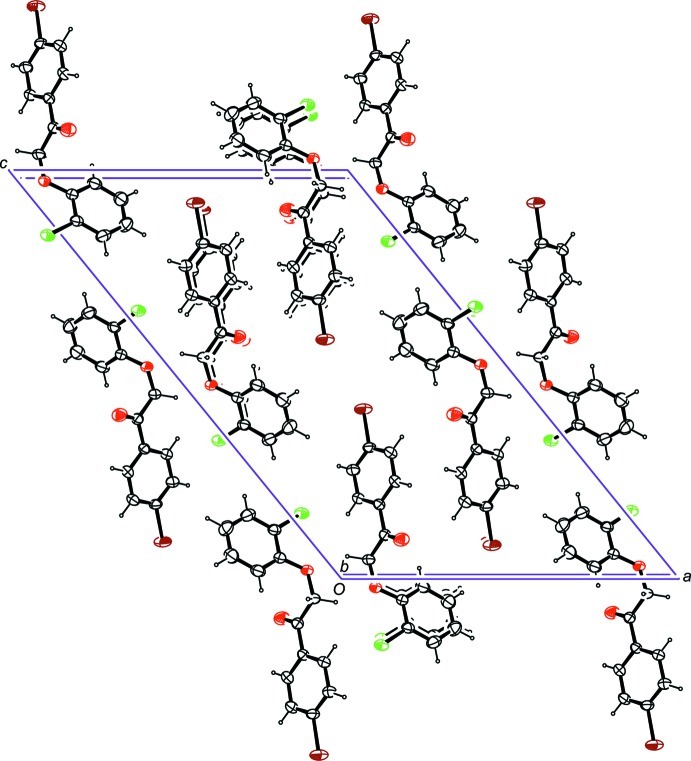
Molecular packing of the title compound, viewed along [0 1 0] (anisotropic displacement ellipsoids drawn at 50% probability level).

## supplementary crystallographic information

### Comment

Derivatives of phenoxyacetic acid are among the most potent fungicides. They demonstrate a variety of biological properties such as antimycobacterial (Ali & Shaharyar, 2007), anti-inflammatory and antioxidant (Kunsch *et al.*, 2005), antibacterial (Iqbal *et al.*, 2007), analgesic (Sato *et al.*, 2002), diuretic (Kitagawa *et al.*, 1991; Bicking *et al.*, 1976) and growth regulatory (Osborne *et al.*, 1955) activity. In continuation of our ongoing interest in bioactive compounds, the title compound was synthesized and its crystal structure was determined.

The O–C–C–O dihedral angle is found at -0.8 (4) °. A statistics of values for dihedral angles of comparable compounds whose molecular and crystal structure has been deposited with the CSD (Allen, 2002) shows that this eclipsed conformation is considerably smaller than the average angle found for the majority of these compounds. The least-squares planes defined by the carbon atoms of the two aromatic moieties enclose an angle of 71.31 (17) ° (Fig. 1 and Fig. 2).

In the crystal, intermolecular C–H···O contacts whose range falls by more than 0.1 Å below the sum of van-der-Waals radii of the atoms participating are apparent. These are supported by one of the hydrogen atoms of the methylene group as well as the hydrogen atom in *ortho* position to the oxygen atom on the chlorophenyl moiety as donors and have the ketonic oxygen atom as acceptor. These connect the molecules to chains along the crystallographic *b* axis. Information about metrical parameters as well as the symmetry of these contacts is summarized in Table 1. In terms of graph-set analysis (Etter *et al.*, 1990; Bernstein *et al.*, 1995), the descriptor for these contacts is *C*^1^_1_(4)*C*^1^_1_(7) on the unary level. In addition, a dispersive Br···Br contact (3.583 (5) Å) was detected. The shortest intercentroid distance between two aromatic systems was measured at 4.554 (2) Å and is present between the chlorinated phenyl moiety and its symmetry-generated equivalent as well as between the brominated phenyl moiety and its symmetry-generated equivalent (Fig. 3). This value is in agreement with the length of cell axis *b*.

The packing of the title compound in the crystal structure is shown in Figure 4.

### Experimental

A mixture of 2-bromo-1-(4-bromophenyl)ethanone (200 mg, 0.00072 mol), potassium carbonate (44.3 mg, 0.00079 mol) and 4-chlorophenol (92.56 mg, 0.00072 mol) in DMF (10 ml) was stirred at room temperature for 2 h. On cooling, colourless needle-shaped crystals of the title compound begin to separate. These were collected by filtration and recrystallized from ethanol, yield: 213.6 mg (91.2%).

### Refinement

Carbon-bound H atoms were placed in calculated positions (C–H 0.95 Å for aromatic carbon atoms and C–H 0.99 Å for methylene groups) and were included in the refinement in the riding model approximation, with *U*_iso_(H) set to 1.2*U*_eq_(C).

### Crystal data

**Table:**

C_14_H_10_BrClO_2_	*F*(000) = 648
*M**_r_* = 325.58	*D*_x_ = 1.690 Mg m^−^^3^
Monoclinic, *P*2_1_/*c*	Melting point = 388–387 K
Hall symbol: -P 2ybc	Mo *K*α radiation, λ = 0.71073 Å
*a* = 15.2653 (8) Å	Cell parameters from 8161 reflections
*b* = 4.5541 (2) Å	θ = 2.7–28.2°
*c* = 23.7336 (9) Å	µ = 3.41 mm^−^^1^
β = 129.135 (2)°	*T* = 200 K
*V* = 1279.80 (10) Å^3^	Needle, colourless
*Z* = 4	0.36 × 0.07 × 0.06 mm

### Data collection

**Table:**

Bruker APEXII CCD diffractometer	3040 independent reflections
Radiation source: fine-focus sealed tube	1953 reflections with *I* > 2σ(*I*)
Graphite monochromator	*R*_int_ = 0.076
φ and ω scans	θ_max_ = 28.0°, θ_min_ = 2.2°
Absorption correction: multi-scan (*SADABS*; Bruker, 2008)	*h* = −20→17
*T*_min_ = 0.377, *T*_max_ = 0.811	*k* = −5→6
15447 measured reflections	*l* = −28→31

### Refinement

**Table:**

Refinement on *F*^2^	Primary atom site location: structure-invariant direct methods
Least-squares matrix: full	Secondary atom site location: difference Fourier map
*R*[*F*^2^ > 2σ(*F*^2^)] = 0.041	Hydrogen site location: inferred from neighbouring sites
*wR*(*F*^2^) = 0.125	H-atom parameters constrained
*S* = 1.02	*w* = 1/[σ^2^(*F*_o_^2^) + (0.0791*P*)^2^ + 0.0906*P*] where *P* = (*F*_o_^2^ + 2*F*_c_^2^)/3
3040 reflections	(Δ/σ)_max_ = 0.001
163 parameters	Δρ_max_ = 0.65 e Å^−^^3^
0 restraints	Δρ_min_ = −1.04 e Å^−^^3^

### Fractional atomic coordinates and isotropic or equivalent isotropic displacement parameters (Å^2^)

**Table:**

	*x*	*y*	*z*	*U*_iso_*/*U*_eq_	
Br1	0.47277 (3)	0.61050 (9)	0.417783 (13)	0.05712 (18)	
Cl1	−0.03928 (6)	−0.09989 (17)	−0.16076 (3)	0.0401 (2)	
O1	0.07748 (15)	0.1858 (5)	−0.02338 (8)	0.0382 (5)	
O2	0.27246 (18)	−0.0310 (6)	0.09698 (10)	0.0438 (5)	
C1	0.1230 (2)	0.3184 (8)	0.04422 (12)	0.0369 (7)	
H1A	0.0676	0.3028	0.0528	0.044*	
H1B	0.1366	0.5294	0.0424	0.044*	
C2	0.2335 (2)	0.1727 (7)	0.10682 (12)	0.0306 (6)	
C11	0.1262 (2)	0.2570 (7)	−0.05457 (12)	0.0324 (7)	
C12	0.0776 (2)	0.1275 (7)	−0.12175 (12)	0.0316 (6)	
C13	0.1196 (3)	0.1825 (8)	−0.15782 (14)	0.0428 (8)	
H13	0.0856	0.0939	−0.2036	0.051*	
C14	0.2109 (3)	0.3659 (8)	−0.12732 (16)	0.0483 (9)	
H14	0.2412	0.4013	−0.1515	0.058*	
C15	0.2582 (3)	0.4979 (9)	−0.06159 (15)	0.0453 (8)	
H15	0.3198	0.6292	−0.0413	0.054*	
C16	0.2173 (2)	0.4421 (8)	−0.02466 (14)	0.0403 (7)	
H16	0.2520	0.5310	0.0212	0.048*	
C21	0.2890 (2)	0.2917 (7)	0.18133 (11)	0.0290 (6)	
C22	0.3935 (2)	0.1744 (8)	0.23899 (13)	0.0373 (7)	
H22	0.4271	0.0270	0.2298	0.045*	
C23	0.4487 (2)	0.2704 (8)	0.30945 (13)	0.0398 (7)	
H23	0.5207	0.1938	0.3485	0.048*	
C24	0.3973 (2)	0.4787 (8)	0.32182 (12)	0.0361 (7)	
C25	0.2928 (2)	0.5983 (7)	0.26559 (13)	0.0379 (7)	
H25	0.2582	0.7413	0.2751	0.046*	
C26	0.2403 (2)	0.5033 (8)	0.19527 (12)	0.0332 (6)	
H26	0.1696	0.5854	0.1561	0.040*	

### Atomic displacement parameters (Å^2^)

**Table:**

	*U*^11^	*U*^22^	*U*^33^	*U*^12^	*U*^13^	*U*^23^
Br1	0.0536 (2)	0.0768 (4)	0.02415 (17)	−0.00832 (17)	0.01652 (15)	−0.01059 (11)
Cl1	0.0349 (3)	0.0493 (6)	0.0267 (3)	−0.0069 (3)	0.0150 (3)	−0.0092 (2)
O1	0.0282 (8)	0.0587 (16)	0.0232 (8)	−0.0105 (9)	0.0142 (7)	−0.0105 (8)
O2	0.0493 (12)	0.0416 (16)	0.0369 (10)	0.0035 (11)	0.0255 (9)	−0.0058 (9)
C1	0.0286 (12)	0.054 (2)	0.0227 (10)	0.0008 (13)	0.0136 (9)	−0.0059 (11)
C2	0.0279 (11)	0.036 (2)	0.0276 (11)	−0.0055 (12)	0.0171 (9)	−0.0044 (10)
C11	0.0263 (11)	0.043 (2)	0.0243 (10)	0.0033 (12)	0.0143 (9)	0.0022 (10)
C12	0.0290 (12)	0.035 (2)	0.0242 (10)	0.0031 (11)	0.0134 (9)	0.0024 (9)
C13	0.0496 (16)	0.049 (2)	0.0318 (12)	0.0052 (15)	0.0265 (12)	0.0039 (12)
C14	0.0552 (19)	0.054 (3)	0.0468 (16)	0.0000 (16)	0.0372 (15)	0.0092 (13)
C15	0.0404 (15)	0.046 (2)	0.0441 (15)	−0.0032 (15)	0.0242 (13)	0.0077 (14)
C16	0.0327 (13)	0.047 (2)	0.0289 (12)	−0.0016 (13)	0.0134 (11)	−0.0006 (11)
C21	0.0240 (11)	0.0334 (19)	0.0255 (10)	−0.0054 (11)	0.0137 (9)	−0.0034 (10)
C22	0.0246 (11)	0.050 (2)	0.0313 (12)	0.0016 (12)	0.0148 (10)	0.0000 (11)
C23	0.0249 (11)	0.055 (2)	0.0288 (11)	0.0004 (13)	0.0121 (9)	0.0036 (12)
C24	0.0320 (12)	0.050 (2)	0.0208 (10)	−0.0104 (13)	0.0141 (10)	−0.0063 (10)
C25	0.0343 (13)	0.047 (2)	0.0276 (12)	−0.0023 (13)	0.0171 (10)	−0.0071 (10)
C26	0.0265 (12)	0.041 (2)	0.0256 (11)	−0.0014 (12)	0.0133 (9)	−0.0028 (10)

### Geometric parameters (Å, º)

**Table:**

Br1—C24	1.887 (2)	C14—H14	0.9500
Cl1—C12	1.740 (3)	C15—C16	1.383 (4)
O1—C11	1.379 (3)	C15—H15	0.9500
O1—C1	1.419 (3)	C16—H16	0.9500
O2—C2	1.202 (4)	C21—C26	1.378 (4)
C1—C2	1.526 (4)	C21—C22	1.396 (4)
C1—H1A	0.9900	C22—C23	1.385 (4)
C1—H1B	0.9900	C22—H22	0.9500
C2—C21	1.499 (3)	C23—C24	1.376 (5)
C11—C16	1.380 (4)	C23—H23	0.9500
C11—C12	1.395 (3)	C24—C25	1.391 (4)
C12—C13	1.379 (4)	C25—C26	1.388 (3)
C13—C14	1.375 (5)	C25—H25	0.9500
C13—H13	0.9500	C26—H26	0.9500
C14—C15	1.376 (5)		
			
C11—O1—C1	117.5 (2)	C14—C15—H15	119.5
O1—C1—C2	111.6 (2)	C16—C15—H15	119.5
O1—C1—H1A	109.3	C11—C16—C15	119.9 (3)
C2—C1—H1A	109.3	C11—C16—H16	120.1
O1—C1—H1B	109.3	C15—C16—H16	120.1
C2—C1—H1B	109.3	C26—C21—C22	119.2 (2)
H1A—C1—H1B	108.0	C26—C21—C2	123.2 (2)
O2—C2—C21	121.9 (2)	C22—C21—C2	117.6 (3)
O2—C2—C1	121.7 (2)	C23—C22—C21	120.7 (3)
C21—C2—C1	116.5 (2)	C23—C22—H22	119.6
O1—C11—C16	125.3 (2)	C21—C22—H22	119.6
O1—C11—C12	115.8 (2)	C24—C23—C22	118.7 (2)
C16—C11—C12	118.9 (2)	C24—C23—H23	120.6
C13—C12—C11	120.8 (3)	C22—C23—H23	120.6
C13—C12—Cl1	120.0 (2)	C23—C24—C25	121.9 (2)
C11—C12—Cl1	119.2 (2)	C23—C24—Br1	118.72 (19)
C14—C13—C12	119.9 (3)	C25—C24—Br1	119.4 (2)
C14—C13—H13	120.1	C26—C25—C24	118.3 (3)
C12—C13—H13	120.1	C26—C25—H25	120.9
C13—C14—C15	119.6 (3)	C24—C25—H25	120.9
C13—C14—H14	120.2	C21—C26—C25	121.1 (2)
C15—C14—H14	120.2	C21—C26—H26	119.4
C14—C15—C16	121.0 (3)	C25—C26—H26	119.4
			
C11—O1—C1—C2	−77.1 (3)	C14—C15—C16—C11	−1.5 (6)
O1—C1—C2—O2	−0.8 (4)	O2—C2—C21—C26	−172.8 (3)
O1—C1—C2—C21	−179.2 (2)	C1—C2—C21—C26	5.6 (4)
C1—O1—C11—C16	1.5 (4)	O2—C2—C21—C22	5.4 (4)
C1—O1—C11—C12	−178.6 (3)	C1—C2—C21—C22	−176.2 (3)
O1—C11—C12—C13	−179.8 (3)	C26—C21—C22—C23	−0.6 (4)
C16—C11—C12—C13	0.1 (4)	C2—C21—C22—C23	−178.9 (3)
O1—C11—C12—Cl1	1.5 (4)	C21—C22—C23—C24	1.6 (5)
C16—C11—C12—Cl1	−178.6 (2)	C22—C23—C24—C25	−1.1 (5)
C11—C12—C13—C14	0.3 (5)	C22—C23—C24—Br1	−179.8 (2)
Cl1—C12—C13—C14	179.0 (3)	C23—C24—C25—C26	−0.3 (5)
C12—C13—C14—C15	−1.3 (5)	Br1—C24—C25—C26	178.4 (2)
C13—C14—C15—C16	1.9 (6)	C22—C21—C26—C25	−0.8 (4)
O1—C11—C16—C15	−179.6 (3)	C2—C21—C26—C25	177.3 (3)
C12—C11—C16—C15	0.5 (5)	C24—C25—C26—C21	1.3 (5)

### Hydrogen-bond geometry (Å, º)

**Table:**

*D*—H···*A*	*D*—H	H···*A*	*D*···*A*	*D*—H···*A*
C1—H1*B*···O2^i^	0.99	2.57	3.457 (4)	149
C16—H16···O2^i^	0.95	2.57	3.425 (4)	150

Symmetry code: (i) *x*, *y*+1, *z*.
